# Foramen magnum meningioma’s management: the experience of the department of neurosurgery in Marrakesh

**DOI:** 10.11604/pamj.2017.26.42.10838

**Published:** 2017-01-30

**Authors:** Farouk Hajhouji, Mohammed Lmejjati, Khalid Aniba, Mehdi Laghmari, Houssine Ghannane, Said Ait Benali

**Affiliations:** 1 Department of Neurosurgery, Mohammed the sixth University Hospital, Marrakesh, Morocco

**Keywords:** Tumors, foramen magnum, meningioma, MRI, surgery

## Abstract

Our study is a retrospective analysis of the clinical data, surgical outcomes, histological finding and prognosis of foramen magnum meningiomas through a serie of 8 cases operated at the department of neurosurgery at Mohammed VI medical university hospital, Marrakesh. From January 2002 to December 2015. There were 3 male and 5 female patients (mean age, 46.75 years). Cervico-occipital pain (100%) and motor deficit (100%) were the most common presenting symptoms. MRI was the most appropriate diagnostic tool in visualizing tumors of this region. All operations were performed by the posterior approach and gross total resection was achieved in 7 cases. Surgical mortality was 20%. 3 other patients had complications like CSF leak (25%), meningitis (12,5%) and transient worsening of neurological deficit (12.5%) but made neurological recovery later. Foramen magnum meningiomas have long been regarded as difficult lesions both in terms of diagnosis and management. However, with the availability of MR imaging, newer surgical techniques and skull base exposures, the excision of these lesions is becoming easier and safer.

## Introduction

Meningiomas of the foramen magmun (FMM) account for 0.3% to 3.2% of all meningiomas, between 4.2% and 20% of all posterior fossa meningiomas and 8.6% of all spinal meningiomas [1]. FMM have been defined by george as tumors arising anteriorly from the inferior third of the clivus to the superior edge of the C2 body, laterally from the jugular tubercle to the C2 laminae, and posteriorly from the anterior border of the occipital squama to the spinal process of C2 [2]. FMM is one of the most challenging types of meningioma to treat because a large number of vital neurovascular structures, which are sensitive to injury, are crowded together in this deeply hidden central area. The intent of this paper is to provide an overview of the clinical data, surgical outcomes, histological finding and prognosis of FMM through a serie of 8 cases operated at the department of neurosurgery at Mohammed VI medical university hospital, Marrakesh.

## Methods

Between january 2002 and decembre 2015 eight patients were operated for FMM at the department of neurosurgery at mohammed VI medical university hospital, marrakesh, Morocco. Medical charts and records were reviewed retrospectively to determine demograophic data, clinical findings, morphological assessment, surgical approaches and outcomes.

## Results

**Figure 1:**
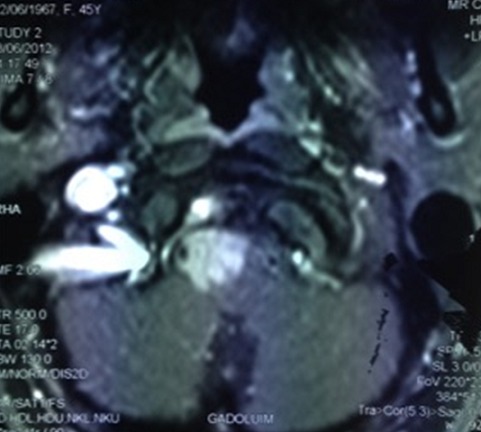
Axial T1 weighted MR image shows an anterolateral foramen magnum meningioma

**Figure 2:**
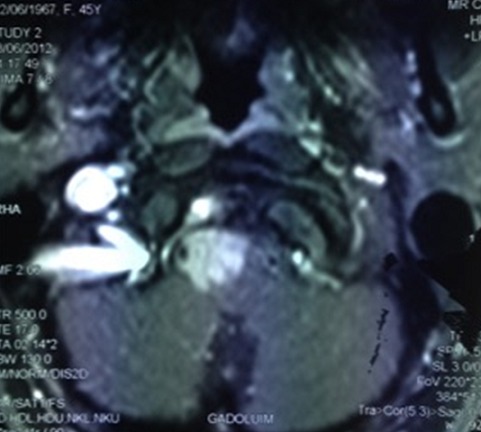
Axial T1 weighted MR image shows the VA encasement by a foramen magnum meningioma

**Figure 3:**
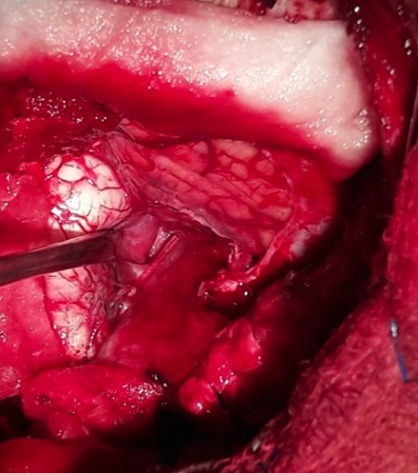
Peroperative photograph shows the microsurgical removal of foramen magnum meningioma trough a midline suboccipital approach

**Figure 4:**
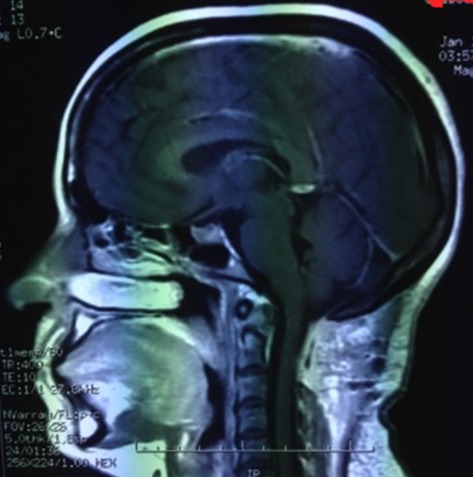
Postoperative T1 Weighted sagittal MRI shows total removal of the lesion

Between January 2002 and December 2015, eight patients were diagnosed with foramen magnum meningiomas. 3(37.5%) were male and 5 (62.5%) were female. The median age was 46.75 (ranging from 23 to 60) The mean duration of symptoms was 13 months (ranging from 4 to 36 months). Presenting signs and symptoms were headache and neck pain in all patients (100%), difficulty in swallowing in 2 patients (25%), gait ataxia in 2 patient (25%) brachalgia and arm paresthesias in 2 patients (25%), monoparesis in one patient (12.5%), hemiparesis in 2 patients (25%) and quadriparesis in 5 patients (62.5%). The mean preoperative KPS was 72.5% (ranging from 60% to 80%). 5/8 (62.5%) of the tumors were located anterolateral (Figure 1), 3/8 were anterior. The mean maximum diameter of the tumors on MRI was 2.75 cm. VA encasement was obeserved in one patient (Figure 2). Posterior midline approach was performed in all cases (Figure 3). Gross-total removal was achieved in 7 of these lesions (87.5%) (Figure 4) and subtotal resection was achieved in one patient who had VA encasement. surgical mortality was observed in two patients (25%) who had postoperative pneuomnia due to swallowing difficulties. CSF fistula occurred in 2 (25%), One patient of them developed meningitis (12.5%) and was successefully treated with antibiotics. Transient worsening of neurological deficit was seen in one patient (12.5%) which spontaneously recovered after two months. All cases were WHO grade I meningiomas. 75% of the tumors were meningothelial, 12.5 % was transitional and 12.5% was psammomatous. Patients were followed for a median of 20 months (range: 3-48 months ). At the latest follow up,6 patients were alive and functionally independent. Mean Karnofsky score was 85% at the latest follow up. No recurrences were observed.

## Discussion

The foramen magnum contains several critical neuroanatomical and vascular structures of which the surgeon must be aware. The neural structures include the cerebellar tonsils, inferior vermis, fourth ventricle, caudal aspect of the medulla, lower cranial nerves (9^th^-12^th^), rostral aspect of the spinal cord, and upper cervical nerves (C1 and C2). arterial structures include the VAs, PICAs, anterior and posterior spinal arteries, and the meningeal branches of the vertebral, external, and internal carotid arteries [3]. Meningiomas occur three times more often than neurinomas, which are the next most common type of benign tumors among those that occur at the foramen magnum. In addition to neurinomas, the differential diagnosis of a foramen magnum meningioma can include dermoids, epidermoids, teratomas, lipomas, hemangioblastomas, cavernous angiomas, giant thrombosed aneurysms of the vertebral artery, intramedullary cervical spinal cord tumors, and syringomyelia [1]. The clinical presentation of patients with FM lesions varies greatly and can mimic many other neurological disorders including multiple sclerosis, cervical spondylosis, and amyotrophic lateral sclerosis. The clinical course is slowly progressive, leading to dysesthesia, asymmetric motor weakness, gait ataxia, and a relatively less common lower cranial nerve palsies [4, 5]. MRI is the modality of choice for defining FMM, It clearly delineates the exact tumor size, location, site of dural attachment, and relation to vascular and neural structures; MRI also provides an opportunity to assess the consistency and vascularity of the tumor [5]. The foramen magnum can be approached via anterior, lateral, and posterior approaches. The anterior transoral approach is rarely conducted to reach intradural lesions such as meningiomas because of problems with dural repair, risk of CSF leakage, and meningitis [3]. Tumors situated posterior or posterolateral to the spinal cord or the brainstem can be safely resected via a posterior midline suboccipital approach combined with C-1 laminectomy [6]. It’s a classic approach wich is familiar to most neurosurgeons [3] and carries a lower morbidity rate than skull base approaches [7]. Tumors situated anteriorly may be accessed with the far-lateral approach described by Heros [8] for VA aneurysms, or the extreme lateral modification described by George et al. [9]. The most devastating complications related to this approach are lower cranial nerve palsies and vertebral artery injury [10]. In our case as few other series [5, 7, 11, 12], only the posterior midline approach was used. According to BYDON [6], GTR can be achieved in 61-100% of the cases. Also vertebral artery involvement significantly affected the rate of radical resection [6].

## Conclusion

According to our experience, we think that, the conventional suboccipital midline approach with C1 laminectomy is sufficient to perform safely the adequate microsurgical removal of foramen magnum meningiomas.

### What is known about this topic

- Meningiomas located in the Foramen magnum are quite uncommon;
- Their surgical treatments remain a technical challenge;
- The far lateral approach is widely considered the gold standard approach by many skull base surgeons.

### What this study adds

- The conventional suboccipital midline approach can safely achieve a total resection of foramen magnum meningiomas;
- A good knowledge of the regional surgical anatomy is sinequanone to achieve a total resection and avoid iatrogenic lesions of the VA and low cranial nerves.
